# Small-Nerve Fibre Pathology in Metabolic Dysfunction-Associated Steatotic Liver Disease Is Associated with Liver Fat/Inflammation and Improves with Weight Loss

**DOI:** 10.3390/nu18193242

**Published:** 2026-10-01

**Authors:** Alex E. Henney, Azlinda Hamid, Jamie Burgess, David R. Riley, Barbara Fielding, Anne Marshall, Khansa Makawi, Abdel-Naser Elzouki, Einas Elgassim, Graham J. Kemp, Rayaz A. Malik, Uazman Alam, Daniel J. Cuthbertson

**Affiliations:** 1 Department of Cardiovascular and Metabolic Medicine, University of Liverpool, Liverpool L69 7ZX, UK; ahenney@liverpool.ac.uk (A.E.H.); jamie.burgess@liverpool.ac.uk (J.B.); david.riley@liverpool.ac.uk (D.R.R.); dan.cuthbertson@liverpool.ac.uk (D.J.C.); 2 Metabolism and Nutrition Research Group, Liverpool University Hospitals NHS Foundation Trust, Liverpool L7 8YE, UK; azlinda.hamid@liverpool.ac.uk; 3 Liverpool Centre for Cardiovascular Sciences, University of Liverpool, Liverpool University Hospitals NHS Foundation Trust, Liverpool L7 8YE, UK; 4 Pain Research Institute, University of Liverpool, Liverpool L9 7AL, UK; anne.marshall@liverpool.ac.uk; 5 Department of Musculoskeletal and Ageing Science, Institute of Life Course and Medical Sciences, University of Liverpool, Liverpool L69 7ZX, UK; 6 Department of Nutritional Sciences, University of Surrey, Guildford GU2 7XH, UK; b.fielding@surrey.ac.uk; 7 Department of General Internal Medicine, Hamad Medical Corporation, Doha P.O. Box 3050, Qatar; kmakawi@hamad.qa (K.M.); aelzouki@hamad.qa (A.-N.E.); 8 Department of Medicine, Weill Cornell Medicine-Qatar, Qatar Foundation, Doha P.O. Box 34110, Qatar; eie2002@qatar-med.cornell.edu; 9 Institute of Life Course and Medical Sciences, University of Liverpool, Liverpool L69 7ZX, UK; gkemp@liverpool.ac.uk; 10 Division of Research, Weill Cornell Medicine, Doha P.O. Box 24144, Qatar; ram2045@qatar-med.cornell.edu; 11 Centre for Musculoskeletal Research, Faculty of Biology Medicine and Health, The University of Manchester, Manchester M13 9PL, UK

**Keywords:** MASLD, obesity, neuropathy, metabolic syndrome, magnetic resonance imaging

## Abstract

**Background:** Metabolic dysfunction-associated steatotic liver disease (MASLD) is associated with multiple extrahepatic complications, but its relationship with peripheral neuropathy, even without co-existing type 2 diabetes (T2D), remains poorly understood. Furthermore, to what extent addressing liver and metabolic health would be associated with improvements in small nerve fibre morphology (that mediate neuropathy) is unknown. We investigated the relationship between liver health and small nerve fibre morphology in adults with MASLD before and after diet-induced weight loss and improvements in liver health. **Methods:** Adults with MASLD (n = 12) and controls without MASLD or T2D (n = 16) underwent anthropometry, biochemistry, transient elastography (TE) and corneal confocal microscopy (CCM). Participants with MASLD then completed a 12-week low-calorie diet (LCD; Counterweight-Plus, ~850 kcal/day) with repeat assessments including multi-organ magnetic resonance imaging (MRI). TE quantified hepatic steatosis (controlled attenuation parameter, CAP), while MRI measured hepatic and pancreatic fat (proton density fat fraction, PDFF) and fibroinflammation (iron-corrected T1, cT1). Neuropathy was assessed using validated questionnaires and CCM-derived corneal nerve fibre density (CNFD), branch density (CNBD) and fibre length (CNFL). The primary outcome measure was change in liver PDFF. Associations between hepatic and neuropathy measures were assessed using correlation and multivariable linear regression. **Results:** Compared with controls, MASLD participants had higher liver fat and metabolic dysfunction, with reduced CNFD (25.7 vs. 28.4 fibres/mm^2^, *p* = 0.03) and CNFL (14.1 vs. 17.1 mm/mm^2^, *p* = 0.02). In multivariable regression, TE-derived CAP was independently associated with CNFD (β −0.03 (95% CI −0.06, −0.01), *p* < 0.05), while total cholesterol was associated with CNFL (β −1.54 (95% CI −2.81, −0.27), *p* < 0.02). After LCD, participants lost 12.5 kg with reductions in metabolic syndrome components (*p* = 0.04) and liver fat (ΔPDFF −5.0%, *p* = 0.03). Neuropathic symptoms improved and CNFD increased (+1.87 fibres/mm^2^, *p* = 0.05). Reduction in liver fat correlated with increased CNFL (r = −0.63, *p* = 0.03), while change in hepatic fibroinflammation (ΔcT1) correlated with neuropathy disability (r = 0.62, *p* = 0.04) and small-fibre symptom scores (r = 0.47, *p* = 0.05). **Conclusions:** These exploratory findings suggest that MASLD, even without T2D, may be associated with small nerve fibre abnormalities and neuropathic symptoms. Diet-induced weight loss improved liver, metabolic and neural health in parallel. Larger prospective confirmatory studies are required to more precisely establish this relationship both at baseline and with interventions.

## 1. Introduction

Metabolic dysfunction–associated steatotic liver disease (MASLD) is the most prevalent chronic liver disease globally, affecting 38% of the adult population, and is expected to increase to 55% over the next 15 years [1]. Indeed, the growing prevalence of MASLD and associated complications has prompted multiple European (EASD, EASO, EASL) and US-based (ADA, AASLD, AACE) disease associations to improve the diagnosis and management of this condition [2]. Whilst decompensated liver disease, hepatocellular carcinoma (HCC), and extra-hepatic complications such as cardiovascular disease, type 2 diabetes (T2D) and cancer are widely acknowledged to be independently associated with MASLD [3,4], there is an increasing body of evidence for an association with microvascular complications in patients with diabetes [5] and pre-diabetes [6], independent of glycaemic control [7]. The severity of MASLD and liver fibrosis has been associated with diabetic peripheral neuropathy [8,9,10] and indeed hepatic sympathetic denervation is implicated in the progression of MASLD [11,12,13].

Lifestyle modification, incorporating dietary energy restriction and weight reduction, remains a cornerstone of MASLD management [14]. Clinically meaningful weight loss is associated with improvements in hepatic steatosis and broader metabolic health [15]. Meta-analysis demonstrates the efficacy of very low energy diets (VLEDs) in reducing body weight and intrahepatic lipid content, as determined by magnetic resonance imaging (MRI)/spectroscopy [16], with similar findings in patients with T2D in the DIRECT study [17]. However, whether these hepatic and metabolic benefits are accompanied by improvements in extrahepatic complications remains less well established.

The extent to which weight reduction, or reduction in liver fat content/fibro inflammation, in patients with MASLD, is associated with improvements in the health of other organs, specifically symptoms and peripheral nerve pathology, remains unclear. In patients with obesity, with [18] and without [19] T2D, metabolic bariatric surgery (MBS)-induced weight loss leads to an improvement in neuropathic symptoms and small nerve fibre regeneration, but no change in quantitative sensory testing, autonomic function or neurophysiology.

We compared small-fibre morphology in a cohort with MASLD to controls without steatosis or associated metabolic dysfunction. Furthermore, in the MASLD cohort, we determined the impact of a 3-month low-calorie diet (~850cal/day) on liver fat and fibro inflammation (measured by TE and MRI), and its association with neuropathic symptoms and small nerve fibre regeneration.

## 2. Methods

### 2.1. Study Design and Participants

**Controls without MASLD or T2D**. Sixteen controls without MASLD or T2D, recruited from General Internal Medicine outpatient clinics in Hamad Medical Corporation, underwent anthropometry, biochemistry, transient elastography and CCM to quantify small nerve fibre morphology in the Clinical Trials Unit, Medical Care and Research Centre, Doha, Qatar. These measures were identical to those taken in the MASLD cohort described below, ensuring methodological consistency between groups.

**Intervention study**. The present study reports the primary and secondary outcome measures from analysis of participants enrolled in the CALIBRATE trial, with mechanistic insights explored (ClinicalTrials.gov NCT04786418). CALIBRATE was a single-centre, mechanistic, randomised controlled trial conducted at Aintree University Hospital, Liverpool, UK, between 2020 and 2024, to assess the effects of a low-calorie dietary (LCD) intervention on metabolic, hepatic and neuronal health in adults living with obesity and MASLD.

**Figure 1:**
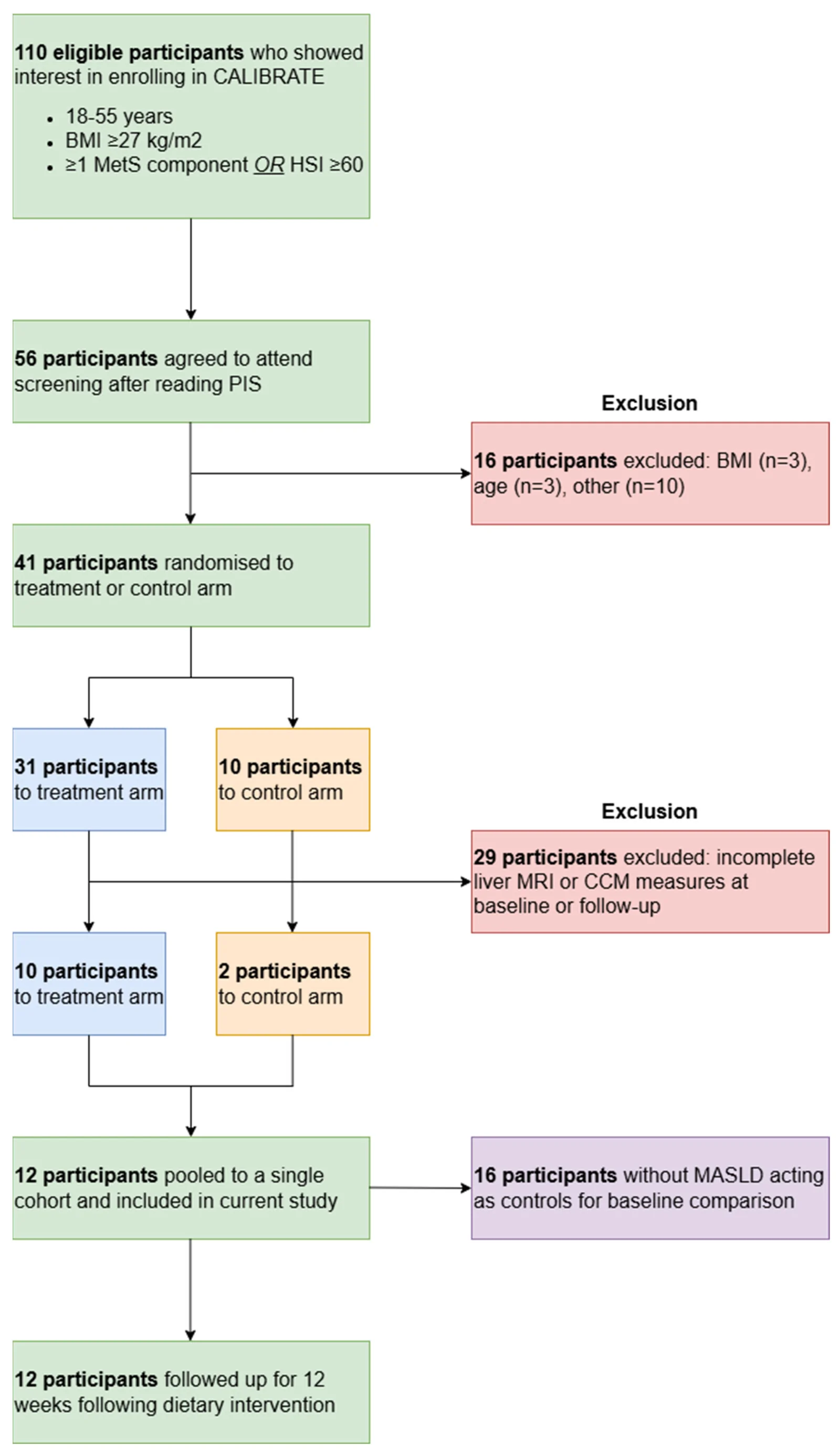
CONSORT diagram demonstrating patient recruitment process for the baseline comparisons between controls and those with MASLD and LCD intervention study in those with MASLD only (CALIBRATE).

Participants were randomised in a 3:1 ratio to either an LCD (Counterweight-Plus programme) intervention (treatment arm) or standard dietitian-led nutritional counselling (control arm). From this cohort, 12 patients with MASLD were recruited (10 in the LCD intervention, 2 in the counselling arm). For the present analysis, data from both arms were pooled to form a single observational cohort, to maximise statistical power and undertake a comprehensive assessment of the relationship between weight loss, TE and MRI-derived indices of hepatic steatosis and fibro-inflammation and CCM-derived measures of small-fibre neuropathy. Both groups received structured dietary interventions, aimed at weight reduction, and underwent identical metabolic and imaging assessments, with the aim of determining the relationship between magnitude of weight loss and multi-organ outcomes rather than comparing treatment efficacy, per se (Figure 1).

The primary outcomes of the trial relate to metabolic and hepatic endpoints, previously unreported. We present a mechanistic analysis of our study findings examining the relationship between hepatic steatosis and small-fibre neuropathy. As these analyses were not formally powered, they should be considered hypothesis-generating.

**Eligibility criteria for the intervention study**. Participants were recruited from primary and secondary care and through local advertising.

*Inclusion criteria.* Participants were aged 18–55 years with body mass index (BMI) ≥ 30 kg/m^2^ (≥27 kg/m^2^ for South Asian or Chinese participants) with evidence of hepatic steatosis (defined as a Fatty Liver Index (FLI) ≥ 60), with at least one metabolic syndrome component (abdominal obesity: waist circumference of ≥102 cm in men and ≥88 cm in women, hypertriglyceridemia: TG ≥ 150 mg/dl (1.695 mmol/L), low HDL-cholesterol: ≥40 mg/dL (1.04 mmol/dL) in men and ≥50 mg/dL (1.30 mmol/dL) in women, high blood pressure: ≥130/85 mmHg, or impaired glucose regulation without diabetes, defined as a high fasting glucose: ≥110 mg/dl (6.1 mmol/L)) [20].

*Exclusion criteria*. Participants were excluded if they were living with type 1 or 2 diabetes, current GLP-1 receptor agonist or SGLT2 inhibitor therapy, prior bariatric surgery, had a recent cardiovascular event, significant alcohol intake (≥21 units/week for men or ≥14 units/week for women), pregnancy or breastfeeding, and contraindication to MRI scanning. Participants with T2D were excluded to minimise confounding from diabetic peripheral neuropathy and to enable investigation of the relationship between MASLD and small nerve fibre morphology in the absence of overt diabetes.

Control participants were recruited as part of an observational study approved by the Hamad Medical Corporation Institutional Review Board (Protocol No. MRC-01-25-260), with reliance acknowledgement from the Weill Cornell Medicine-Qatar Institutional Review Board (IRB No. 25-00023). The study was conducted in accordance with institutional policies and international ethical guidelines, and all participants provided written informed consent prior to participation. The CALIBRATE study has been through the ethics arrangement required by Sponsor (University of Liverpool) and the Health Research Authority (HRA), through the Integrated Research Application System (IRAS) project ID: 270174. This trial is a randomised clinical trial, registered with the ClinicalTrials.gov PRS registry, number NCT04786418, released on 5 March 2021. The study was conducted in accordance with the Declaration of Helsinki, Good Clinical Practice guidelines, and all applicable UK regulatory requirements. All participants provided written informed consent prior to enrolment.

### 2.2. Study Procedures

#### 2.2.1. Baseline Comparison Study Between Controls and MASLD

**Anthropometric and Biochemical Measures**. Body weight, height, waist and hip circumference were measured using calibrated equipment, and BMI calculated as weight (kg)/height (m)^2^. Blood pressure was recorded after 5 min’ rest. Fasting blood samples were analysed for glucose, HbA1c, lipid profile, liver function (alanine and aspartate aminotransferases (ALT, AST), γ-glutamyl transferase (GGT), platelets, albumin), and full blood count.

#### 2.2.2. Imaging

**Transient elastography** was performed using FibroScan^®^ (Echosens, Paris, France) by trained operators according to standard protocols. Liver stiffness measurement (LSM, kPa) was used as a non-invasive marker of hepatic fibrosis and the controlled attenuation parameter (CAP, dB/m) as a surrogate of hepatic steatosis. Measurements were obtained after a minimum 3-h fast, with the appropriate probe (M or XL) selected automatically based on body habitus. Ten valid readings were acquired for each participant, and results were considered reliable when the interquartile range-to-median ratio was <30% for LSM.

**Magnetic Resonance Imaging (MRI)**. MRI studies were performed on a 3.0 T Siemens Prisma scanner using the CoverScan/LiverMultiScan V1^®^ protocol (Perspectum Diagnostics, Oxford, UK). Standardised acquisition included multi-echo gradient-echo sequences for proton density fat fraction (PDFF) quantification and T1 mapping with correction for iron content (iron-corrected T1, cT1). All image analysis was performed centrally by Perspectum Diagnostics, blinded to clinical and biochemical data, with MRI quality control conducted according to manufacturer and site-specific Standard Operating Procedures.

*Liver*. Liver fat (PDFF) was expressed as the percentage of hepatic proton density attributable to fat, while hepatic fibro-inflammatory activity was assessed using cT1 (ms), with higher values indicating greater inflammation and fibrosis.

*Pancreas and kidney*. In addition to the liver, whole-organ metabolic phenotyping of the pancreas and kidneys was undertaken using the Perspectum MR protocol, which applies the same multi-echo PDFF methodology to quantify pancreatic fat fraction and uses T1-based tissue characterisation to assess pancreatic tissue health. Renal cortical T1 mapping was also acquired, providing a non-invasive biomarker of renal tissue integrity and inflammation, as previously validated in metabolic disease cohorts [21].

#### 2.2.3. Neuropathy Measures

*Corneal Confocal Microscopy (CCM)* (Heidelberg Retinal Tomograph III with Rostock Corneal Module, Heidelberg Engineering, Heidelberg, Germany) was used to assess corneal small-nerve fibre morphology in vivo. After topical anaesthesia (0.4% benoxinate), participants were instructed to fixate on a target light while central corneal images were obtained using a 63× objective lens (numerical aperture 0.9). At least six high-quality, non-overlapping central images per participant were captured, and the three best images were analysed. Quantitative analysis used ACCMetrics software V1 (University of Manchester, Manchester, UK) to derive: (i) corneal nerve fibre density (CNFD, fibres/mm^2^), number of main fibres per area; (ii) corneal nerve branch density (CNBD, branches/mm^2^), number of branches per area; and (iii) corneal nerve fibre length (CNFL, mm/mm^2^), total length of all nerve fibres per area. All analyses were performed by trained investigators blinded to participant identity, visit, and study arm. Corneal nerve health was compared to controls without MASLD/T2D, and normative reference ranges from the existing literature [22].

*Neuropathy questionnaires* assessed subjective neuropathic symptoms and signs using validated clinical instruments. The McGill Pain Questionnaire (MPQ) was used to characterise neuropathic pain quality and intensity across sensory and affective dimensions. The Neuropathy Symptom Profile (NSP) provided a structured assessment of positive (pain, paraesthesia) and negative (numbness, sensory loss) symptoms affecting small and large fibres. The PainDETECT Score (PDS) evaluated neuropathic pain components using a patient-completed 9-item questionnaire, with scores ≥19 suggesting a likely neuropathic origin. Finally, the Small Fiber Neuropathy Screening List (SFNSL) was administered to capture autonomic and somatic small-fibre symptoms (e.g., burning, allodynia, thermal dysregulation). The Neuropathy Disability Score (NDS) quantified objective neurological deficits based on examination of vibration perception, pin-prick sensation, temperature discrimination, and ankle reflexes, with higher scores indicating greater impairment. All questionnaires were administered at baseline and 3 months by trained research staff, and composite scores were calculated according to validated scoring algorithms.

**Intervention study in MASLD participants**. Participants allocated to the LCD arm followed the Counterweight-Plus programme, comprising: Total diet replacement (~850 kcal/day) with nutritionally complete formula shakes and soups for 3 months (this was a nutritionally complete formulated diet rather than a selectively nutrient-restricted intervention, designed primarily to achieve substantial energy restriction rather than specifically manipulating individual macronutrients or micronutrients), whilst participants in the standard-care arm received dietetic counselling based on national guidance for gradual weight reduction through caloric restriction (~600 kcal/day deficit), promotion of Mediterranean-style dietary patterns, and physical-activity advice. All participants attended identical study visits and investigations at baseline and follow-up. Those in the control arm received only standard dietitian-led nutritional counselling.

**Outcomes**. The primary outcome was the delta change in hepatic fat content (PDFF) from baseline to 3 months. Secondary outcomes included delta changes in: (i) hepatic fibro-inflammatory activity (cT1, ms); and (ii) CCM-derived small-nerve fibre parameters (CNFD, CNBD, CNFL) and neuropathy questionnaire scores. Exploratory analyses examined associations between hepatic and small nerve fibre morphological changes and between metabolic biomarkers (e.g., insulin resistance) and organ-specific outcomes.

**Statistical Analysis**. Analyses were performed using SPSS version 29 (IBM Corp., Armonk, NY, USA). Data were assessed for normality using the Shapiro–Wilk test and visual inspection of histograms and Q–Q plots. Given the modest sample size and non-normal distribution of several variables, continuous data are presented as mean ±  standard deviation (SD) where normally distributed, or median (interquartile range [IQR]) where non-normally distributed. Categorical variables are expressed as counts (percentages). A two-tailed *p* value < 0.05 was considered statistically significant.

*Baseline comparison*. Clinical, metabolic, imaging and CCM parameters were compared between participants with MASLD and controls using independent samples tests (Welch’s *t*-test for normally distributed data or Mann–Whitney U test for non-normally distributed data).

To explore determinants of small-fibre morphology at baseline across the combined cohort (MASLD and controls), univariate correlations between CCM metrics and metabolic or hepatic variables were assessed using Pearson or Spearman’s correlation coefficients. Variables demonstrating significant or near-significant associations in univariate analyses were subsequently entered into a multivariable linear regression model to identify independent predictors of CCM metrics. Given the overall sample size, the number of covariates included in each model was limited to avoid overfitting. Regression results are presented as unstandardised beta coefficients (β) with 95% confidence intervals (CI), standardised β, and *p* values.

*Pre- and post-LCD intervention*. Changes following the 12-week dietary intervention were assessed using paired analyses (paired *t*-test or Wilcoxon signed-rank test, as appropriate) in the MASLD cohort. Within-participant delta change (Δ) for each parameter was calculated as follow-up minus baseline. Associations between ΔMRI and ΔCCM outcomes, with other covariates, were assessed using Pearson or Spearman correlation coefficients, as appropriate to data distribution. Given the limited number of participants completing follow-up, multivariable regression modelling was not performed for longitudinal analyses, which were considered exploratory and hypothesis-generating. All analyses followed a complete-case approach.

## 3. Results

### 3.1. Baseline Characteristics

*Clinical and demographic characteristics*. All baseline clinical and demographic data are presented in Table 1. Sixteen control participants without MASLD (11 men, 5 women), with a median age of 40 (33, 46) years and twelve participants with MASLD (5 men, 7 women), with a median age of 48 (IQR 40, 53) years were included. Metabolic dysfunction was greater in the MASLD group (median number of MetS components, −3 (2, 4) vs. 2 (1, 3) (*p* < 0.01)). Specifically, MASLD participants were living with more severe overweight/obesity (median BMI-35.7 (35.3, 37.8) vs. 25.3 (22.9, 28.9) kg/m^2^ (*p* < 0.01), body weight-112.7 (87.4, 118.2) vs. 71.0 (56.5, 79.8) kg (*p* < 0.01), waist circumference-114.3 (105.6–120.5) vs. 79.5 (72.5, 86.3) cm, and hip circumference-117.0 (115.3, 126.3) vs. 84.5 (80.0, 90.5) cm (*p* < 0.01)) and had significantly higher fasting glucose (5.1 (4.5, 5.5) vs. 4.2 (4.0, 4.4) mmol/L (*p* < 0.01)), total (5.7 (5.5, 6.3) vs. 4.4 (3.9, 4.8) mmol/L (*p* < 0.01)), and LDL (3.8 (3.3, 4.2) vs. 2.5 (2.1, 2.8) mmol/L (*p* < 0.01), cholesterol.

*Liver health*. Participants with MASLD had significantly worse non-invasive markers of liver disease. Fatty liver index, FLI (88.5 (78.3, 96.8) vs. 18.0 (6.7, 34.7) (*p* < 0.01)) and NAFLD fibrosis scores, NFS (−2.00 (−2.56, −1.34) vs. −2.80 (−3.56, −1.89) (*p* = 0.03)) demonstrated a higher risk of steatosis and fibrosis, respectively; although a NFS of −2.00 in the MASLD participants represents an overall low risk of advanced fibrosis according to validated cut offs [23]. CAP measurements generated via transient elastography were significantly higher in participants with MASLD (316 (262, 343) vs. 209 (197, 236) dB/m (*p* < 0.01)). MRI liver parameters demonstrated evidence of hepatic steatosis (MRI-PDFF 9.4 (3.8, 14.2)%; normal <5%) and mild fibro-inflammation (median cT1 805 (736, 857) ms; normal < 795 ms) (Table 1).

*Pancreas and kidney health*. The median levels of pancreatic fat (pancreas PDFF) and pancreatic and kidney fibro-inflammation (srT1 and cortical T1) were within normal limits in participants with MASLD (Table 1).

**Figure 2:**
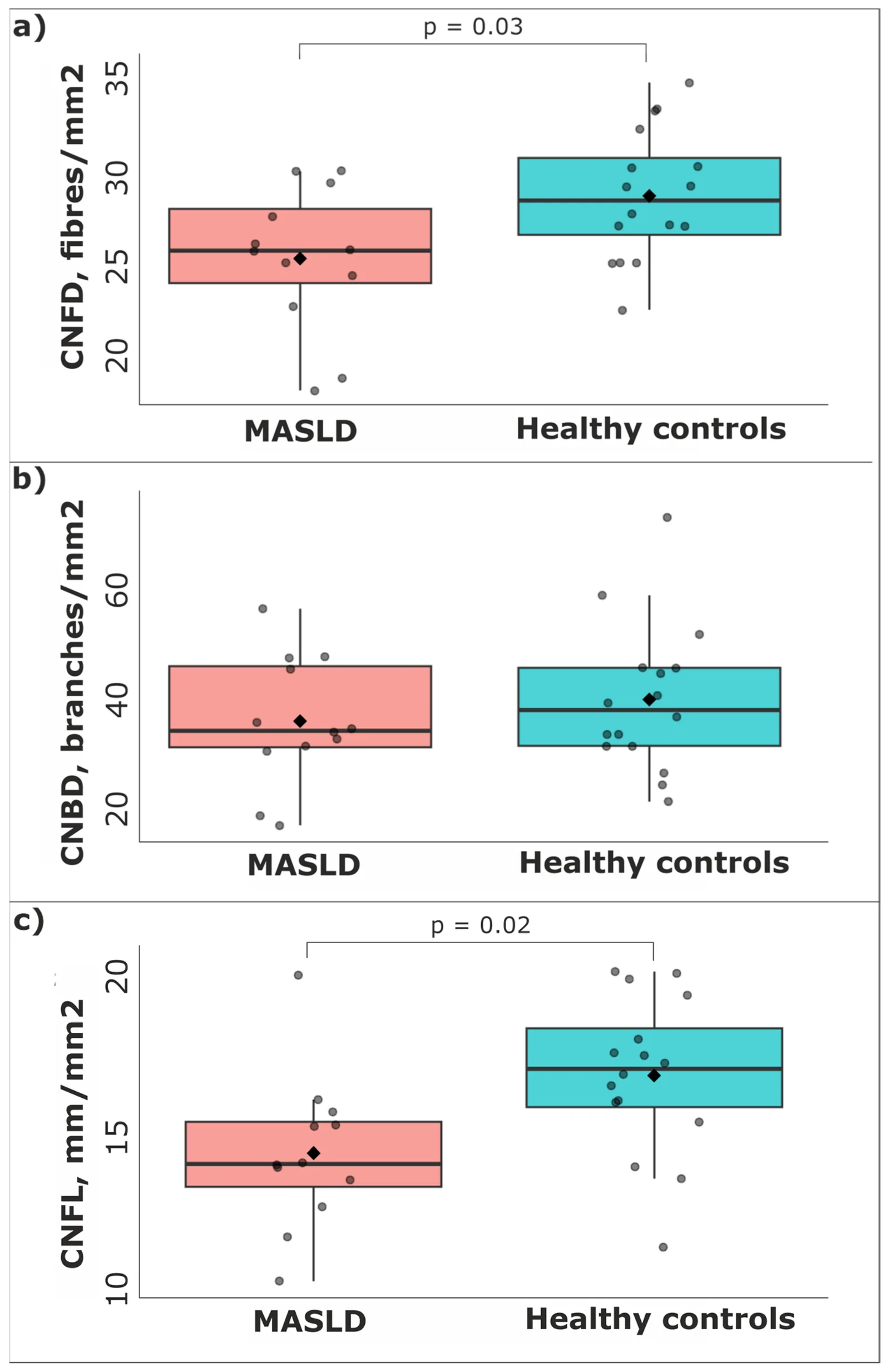
Boxplots demonstrating significantly reduced corneal nerve fibre density and length in patients with MASLD compared to controls. (**a**) corneal nerve fibre density (CNFD); (**b**) corneal nerve branch density (CNBD); (**c**) corneal nerve fibre length (CNFL).

*Nerve health*. Median CNFD (25.7 vs. 28.4 fibres/mm^2^ (*p* = 0.03)) and CNFL (14.1 vs. 17.1 mm/mm^2^ (*p* = 0.02)) were significantly lower in participants with MASLD compared to controls, with no significant difference in CNBD (34.1 vs. 37.4 branches/mm^2^) (Table 1 and Figure 2), consistent with small-fibre loss compared to normative values [22].

### 3.2. Determinants of Small-Fibre Morphology at Baseline

*Univariate analysis* When using data from all participants (MASLD and controls), BMI (r = −0.42 (*p* = 0.03)), body weight (r = −0.53 (*p* < 0.01)), waist (r = −0.45 (*p* = 0.02)), and hip (r = −0.48 (*p* < 0.01)), circumference, negatively correlated with CNFD. BMI (r = −0.40 (*p* = 0.03)), body weight (−0.44 (*p* = 0.02)), and hip circumference (r = −0.44 (*p* = 0.02)) also negatively correlated with CNFL. Total cholesterol negatively correlated with CNFD (r = −0.40 (*p* = 0.04)) and CNFL (r = −0.54 (*p* < 0.01)) and LDL cholesterol negatively correlated with CNFL (r = −0.52 (*p* < 0.01)). FLI correlated negatively with CNFL (r = −0.41 (*p* = 0.04)) and transient elastography-derived CAP correlated negatively with CNFD (r = −0.45 (*p* = 0.02)) and CNFL (r = −0.40 (*p* = 0.04)) (Supplementary Material Table S1).

In participants with MASLD, liver fibro-inflammation (cT1) positively correlated with FLI (r = 0.58; *p* = 0.04). There were significantly inverse correlations between CNFD and BMI (r = −0.64; *p* = 0.02), weight (r = −0.61; *p* = 0.04), waist (r = −0.59; *p* = 0.04) and hip (r = −0.57; *p* = 0.05) circumference, and diastolic blood pressure (r = −0.66; *p* = 0.02). All data is presented in Supplementary Material Tables S2 and S3, with correlation scatter plots presented in Supplementary Material Figure S3.

*Multi-variate analysis*. A multivariable linear regression model, including strongly associated variables (BMI, total cholesterol, transient elastography-derived CAP, and age) demonstrated independent associations between CAP and CNFD (uβ −0.03, 95% CI −0.06, −0.01; sβ −0.45 (*p* < 0.05)), and total cholesterol with CNFL (uβ −1.54, 95% CI −2.81, −0.27; sβ −0.53 (*p* < 0.02)). BMI was not independently associated with any CCM metric (Table 2).

### 3.3. Effects of Dietary Intervention

*Clinical and demographic characteristics* At 3 months, participants achieved significant reductions in body weight (−12.5 kg (−13.6 to −8.4); *p* < 0.01), BMI (−4.0 kg/m^2^ (−5.4 to −3.0); *p* < 0.01), and MetS components (−1 (0, 2); *p* = 0.04); specifically, waist circumference (−12.0 cm (−17.9 to −5.6); *p* = 0.01), and systolic blood pressure (−5 mmHg (−19 to 0); *p* = 0.03) (Table 1).

**Figure 3:**
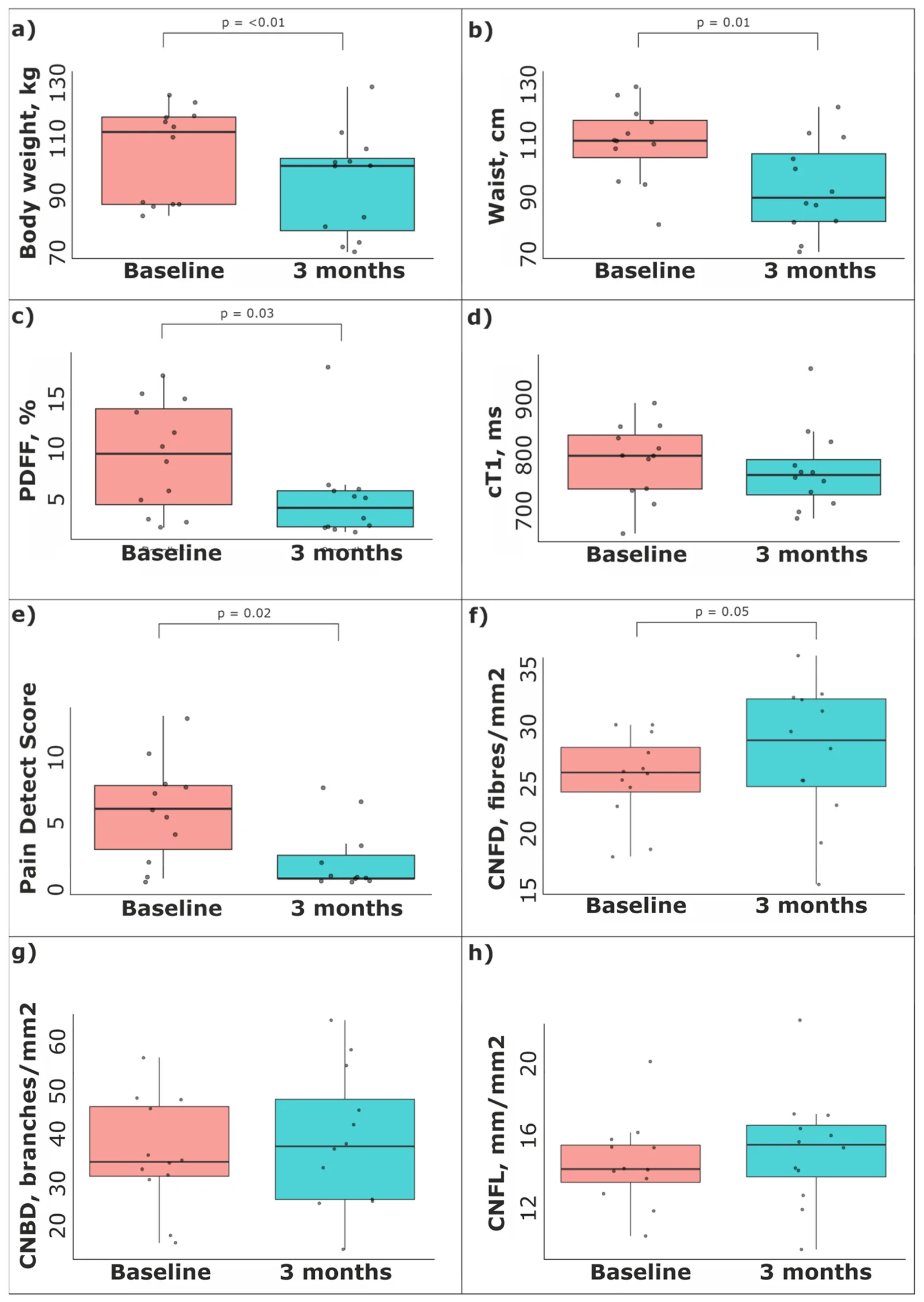
Boxplots demonstrating change in anthropometric, liver imaging (PDFF) and corneal confocal parameters from baseline to 3 months following dietary intervention in those with MASLD. (**a**) body weight in kilograms (kg); (**b**) waist circumference in centimeters (cm); (**c**) magnetic resonance imaging-derived proton density fat fraction (PDFF); (**d**) magnetic resonance imaging-derived-liver iron corrected T1 (cT1); (**e**) Pain Detect Score; (**f**) corneal nerve fibre density (CNFD); (**g**) corneal nerve branch density (CNBD); (**h**) corneal nerve fibre length (CNFL).

**Figure 4:**
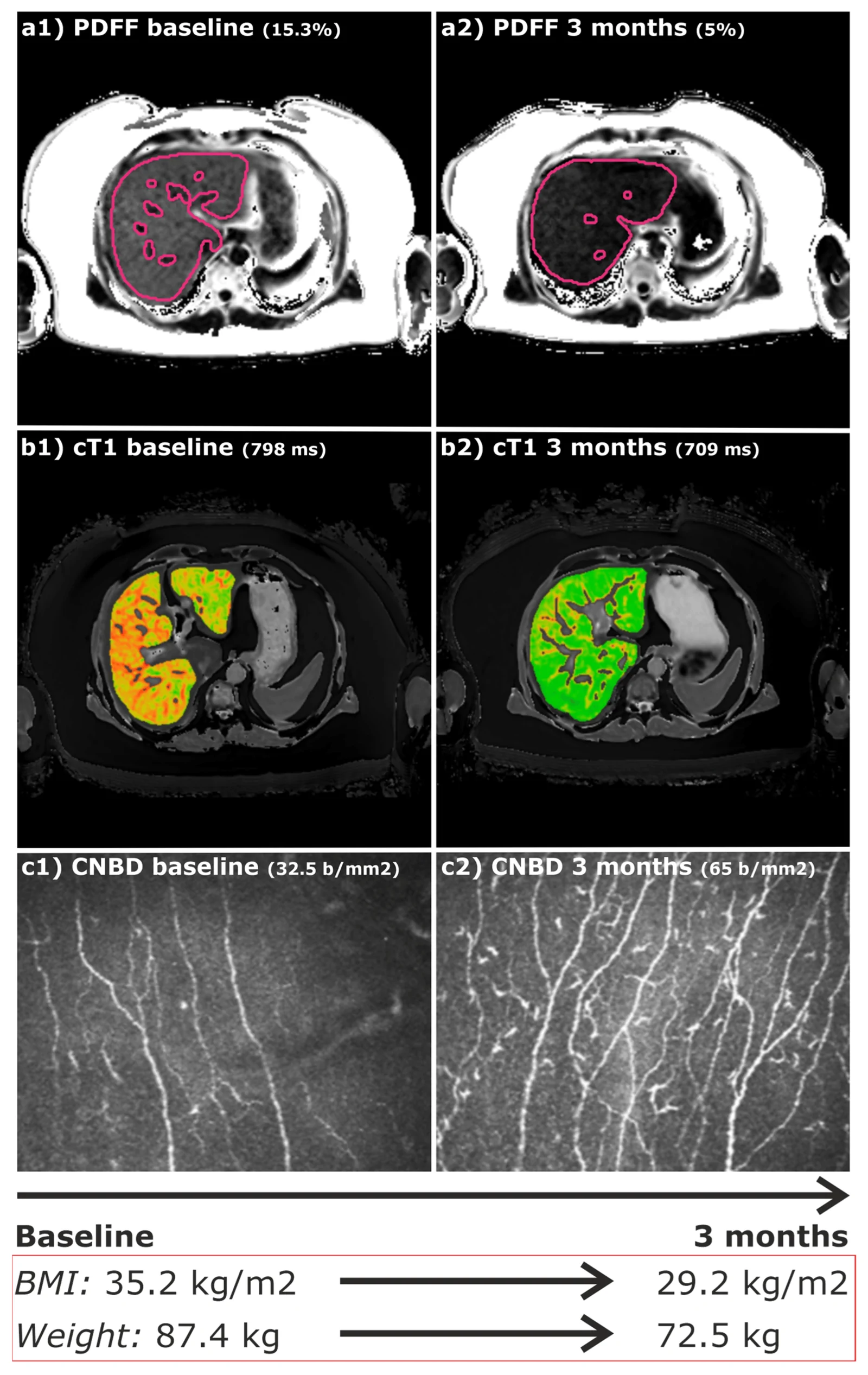
Representative images from a participant following diet-induced weight loss demonstrating improvement in (**a1**,**a2**) liver proton density fat fraction, (**b1**,**b2**) liver-iron corrected T1 (using MRI) and (**c1**,**c2**) corneal nerve branch density (using corneal confocal microscopy), from baseline (**a1**,**b1**,**c1**) to 3 months (**a2**,**b2**,**c2**).

*Liver health* The Fatty Liver Index reduced from 88.5 (78.3–96.8) to 66.4 (38.8–86.5) (*p* = 0.03) (Supplementary Material Figure S1). Hepatic steatosis improved significantly: liver fat (PDFF) decreased from 9.4 (3.8–14.2)% to 4.4 (2.5–6.1)% (*p* = 0.03), while fibro-inflammatory activity (cT1) showed a non-significant trend toward reduction (−30 ms (−76 to 41); *p* = 0.29) (Figure 3 and Figure 4).

*Pancreas and kidney health*. There were no significant changes in pancreatic fat nor in pancreatic and kidney fibro-inflammation (Table 1).

*Neuropathy*. CNFD increased significantly (+1.87 fibres/mm^2^; *p* = 0.05) while CNBD and CNFL showed a numerical but non-significant increase (Figure 3 and Figure 4). Neuropathic symptom burden decreased (Neuropathy Symptom Profile −1.5 (*p* = 0.02), PainDETECT −5.0 (*p* = 0.02), and SFNSL −11.0 (*p* = 0.03) (Supplementary Material Figure S2).

**Associations between changes in liver, metabolic and neural parameters**. There were significant correlations between change in metabolic and liver health with change in neuropathy measures. Reduction in liver fat (ΔMRI-PDFF) was significantly associated with ΔCNFL (r = −0.63, *p* = 0.03) while reduction in liver fibro-inflammation (ΔcT1) correlated with the Neuropathy Disability Score (r = 0.62, *p* = 0.04), and Small Nerve Fibre Screening List (r = 0.47, *p* = 0.05). ΔCNBD correlated with FIB-4 (r = −0.64; *p* = 0.05) while ΔCNFD correlated with diastolic blood pressure (r = −0.72; *p* = 0.01). All data is presented in Supplementary Material Tables S4 and S5, with correlation scatter plots in Supplementary Material Figure S4.

## 4. Discussion

We demonstrate important baseline associations between liver and metabolic health with neural health. We find hepatic steatosis, assessed using Fibroscan-derived CAP, was independently associated with lower nerve fibre density after adjustment for metabolic covariates, suggesting an association beyond overall adiposity. Furthermore, following dietary intervention, weight loss and improvements in liver and metabolic health were accompanied by improvements in neuropathic symptoms and an increase in nerve fibre density (CNFD); changes in CNBD and CNFL were not statistically significant. The correlation between the reduction in MRI-derived hepatic fat and the increase in CNFL suggests that changes in hepatic and neural health may be related.

**Associations of neuropathy with MASLD**. In the current study, although markers of adiposity and dyslipidaemia were associated with CCM parameters on univariate analysis, TE-derived CAP remained independently associated after multivariable adjustment, suggesting that hepatic steatosis itself may contribute directly to small fibre injury rather than merely reflecting overall obesity or its associated metabolic burden. Observational studies have shown that MASLD is independently associated with peripheral neuropathy [8,9,10]. Cross-sectional data from 3029 participants (aged 40–85 years), with MASLD (defined as a FLI score ≥ 30), enrolled in the 1999–2004 National Health and Nutrition Examination Survey (NHANES), found a 44% increased risk of neuropathy (defined by ≥1 insensate area to the monofilament on the foot) after fully adjusting for covariates [8]. Similarly in a study of 264 individuals with T2D from South Korea, liver fibrosis was independently associated with DPN based on the MNSI score, after adjustment for other covariates [10]. In a study of 520 patients with T2D from China, the severity of hepatic steatosis and fibrosis was associated with neuropathy assessed using nerve conduction testing [9]. In our previous retrospective cohort study of almost 300,000 patients with MASLD, we demonstrated a graded association of MetS components with the risk of neuropathy: those with MASLD and one MetS component had a 13-fold increased risk of peripheral neuropathy over 5 years, whereas those with MASLD and four metabolic syndrome components had over a 300-fold increased risk of peripheral neuropathy, with insulin resistance carrying the greatest individual risk of neuropathy [3].

**Improvements in neuropathy with weight loss**. In a study of patients with obesity without T2D, 12 months after bariatric surgery the mean BMI had reduced by 15 kg/m^2^, with a significant improvement in CNFD, CNBD and CNFL and the increase in CNFD correlated with a decrease in triglycerides. The greater increase in CCM measures in this cohort, relative to the current study, may be explained by the greater weight loss associated with bariatric surgery over diet-induced weight loss, and the longer follow-up period (3 vs. 12 months) and the manual quantification of corneal nerve measures [19]. In a study of 20 participants with obesity (65% with T2D), 12 months after bariatric surgery, there was a reduction in BMI and an improvement in CNFD, CNBD and CNFL [24]. A prospective study of 72 participants with obesity, followed for 2 years following dietary intervention showed no significant improvement in intra-epidermal nerve fibre density (IENFD) [25]. However, in a study of patients with T1D undergoing simultaneous pancreas and kidney transplantation, corneal nerve regeneration occurred within 6 months, whilst there was no change in IENFD even at 36 months [26].

Despite recent advances in the pharmacological approach to the management of MASLD with agents such as resmetirom, GLP-1 (± GIP) RAs and SGLT2 inhibitors, weight reduction via lifestyle interventions remains the cornerstone of MASLD management and has been shown to improve hepatic steatosis, inflammation, and fibrosis; most sensitively measured via multi-parametric MRI liver indices [16,27]. In parallel, corneal confocal microscopy has been deployed in studies in patients with obesity and T2D to demonstrate small nerve fibre regeneration after bariatric surgery [19,24], GLP-1 therapy [28] and recently SGLT2 inhibitor therapy [29]. These improvements correlated with improvements in metabolic dysfunction, hyperlipidaemia and systemic inflammation [19,30,31]. Our observation that weight loss was accompanied by improvements in hepatic MRI biomarkers, neuropathic symptoms and CNFD suggests that metabolic improvement may confer multi-organ benefits extending to the peripheral nervous system, although these exploratory findings require confirmation in larger studies.

**Mechanistic insights**. Weight loss in MASLD plausibly initiates neural repair by reducing the hepatic and systemic lipotoxicity that drive insulin resistance, oxidative stress, and inflammatory signalling (TNF-α/JNK), thereby lowering the neurotoxic burden to small fibres [32,33,34,35,36]. As hepatic steatosis regresses, both downstream pro-inflammatory cytokines and mitochondrial stress reduce, diminishing peripheral axonal injury. In parallel, MRI biomarkers capture these hepatic changes: PDFF falls with effective lifestyle weight loss, and cT1 reflects improvement in fibro-inflammatory activity [19,24]. In our cohort, reductions in hepatic steatosis were associated with improvements in small nerve fibre morphology; however, this does not establish a direct causal relationship. Weight loss improves multiple domains of metabolic health including insulin sensitivity, glycaemic control, dyslipidaemia and systemic inflammation, each of which may independently promote small nerve fibre repair. Although the independent baseline association between CAP and CNFD, after adjustment for age, BMI and total cholesterol, suggests an association between hepatic steatosis and small nerve fibre morphology beyond that explained by overall adiposity, the small sample size precluded multivariable or mediation analyses of longitudinal changes. Consequently, improvements in hepatic and neural measures may reflect broader metabolic health improvement, rather than a direct specific effect of reduced hepatic fat. Triglyceride-mediated lipotoxicity may provide a shared mechanistic pathway between MASLD and neuropathy with hypertriglyceridaemia being strongly associated with reduced intra-epidermal nerve fibre density and increased neuropathy risk, independent of glycaemic control [37]. Consistent with this, total cholesterol was independently associated with CNFL in our multivariable analysis. We have also previously shown that hypertriglyceridaemia is associated with corneal small nerve fibre degeneration [38,39,40], and is particularly associated with visceral rather than subcutaneous adiposity [41].

A second, MASLD-relevant pathway is restoration of a pro-regenerative neurotrophic milieu. MASLD is associated with dysregulated trophic signalling; human and experimental data link disease severity to lower IGF-1, which supports axonal growth and Schwann-cell survival and altered NGF/p75^NTR^ expression in relation to the severity of hepatic fibrosis [42,43]. Weight reduction and improvement in hepatic steatosis tend to normalise IGF-1, while neurotrophins such as VEGF and NGF, key drivers of nerve regeneration, promote corneal neurite elongation; whilst VEGF blockade impairs corneal nerve repair [24,43]. These trophic dynamics provide biologic plausibility for corneal nerve regeneration accompanying improvements in hepatic steatosis in our study.

Thirdly, hepatic–autonomic crosstalk may couple liver and nerve outcomes. MASLD exhibits hepatic sympathetic overactivity; in vivo hepatic denervation reduces steatosis and normalises hepatic lipid acquisition pathways; evidence that liver–nerve signalling is bidirectional [11,44]. Emerging work also implicates brain–liver parasympathetic circuits in hepatic lipid handling [45]. Thus, attenuation of hepatic fibro-inflammation may rebalance autonomic tone, improving systemic metabolism and indirectly benefitting small nerve fibres. Available human data suggest a temporal sequence in which hepatic and associated metabolic improvement precede or coincide with small-fibre recovery: effective weight loss reduces liver fat within weeks to months and lowers inflammation; CCM-detected nerve regeneration typically emerges over subsequent months [19,24]. Microvascular/endothelial dysfunction reported in MASLD provides an additional bridge between hepatic inflammation and small-fibre vulnerability, further supporting a liver-to-nerve progression that our ΔPDFF–ΔCNFL and ΔcT1–neuropathy correlations are consistent with [32,33,34,35,36].

**Clinical implications**. These findings have important clinical implications. Peripheral neuropathy is not routinely screened for in MASLD, particularly in individuals without diabetes. Our data suggest that small nerve fibre abnormalities may occur in MASLD and may be potentially modifiable alongside improvements in metabolic and hepatic health. CCM may have potential as a non-invasive research tool for detecting early neural injury and monitoring extrahepatic treatment response in MASLD.

**Strengths and limitations**. The analyses presented here were not powered for neuropathy outcomes, and thus these outcomes should be interpreted as exploratory. Moreover, in this analysis, we provide pooled prospective follow-up of a relatively small cohort of subjects with MASLD incorporating treatment effects of both arms of dietary intervention. This was undertaken due to the small numbers with data completeness for neuropathy and liver imaging measures in the study cohort; in fact, only 2 participants in the control arm had complete MRI liver and CCM data. The small sample size substantially limits the precision and generalisability of these findings, particularly the longitudinal analyses. These findings should be considered hypothesis-generating and require confirmation in larger prospective studies.

Although CNFD increased following dietary intervention, changes in CNBD and CNFL were not statistically significant. The observed associations therefore do not establish causality or demonstrate generalised small nerve fibre regeneration and should be considered hypothesis-generating. Nevertheless, our participants were deeply phenotyped, with detailed measures of liver health (through transient elastography and MRI measures), neuropathic pain, small nerve fibre symptoms and deficits and corneal confocal imaging which have allowed us to identify small nerve fibre regeneration after diet-induced weight loss. We acknowledge that we do not have data on intraepidermal nerve fibre density, a sensitive marker of small fibre neuropathy, nor do we have data on specific micronutrient biomarkers, such as serum Vitamin B12 or Vitamin D levels, and therefore the potential contribution of micronutrient deficiencies to neuropathic measures cannot be excluded. Moreover, physical activity was not systematically quantified and could therefore represent an unmeasured confounder, as changes in habitual activity may have contributed to improvements in metabolic, hepatic and neural outcomes. Finally, our participants with MASLD were predominantly living with steatosis, and only mild fibroinflammation in some cases, and therefore examination of neuropathy outcomes across the entire MASLD spectrum (from steatosis, through steatohepatitis, fibrosis and cirrhosis) would better aid our understanding of this extra hepatic manifestation. Evaluation of neuropathy status in those with advanced hepatic fibrosis or cirrhosis would be of value. Future work should also evaluate whether pharmacological MASLD therapies, including GLP-1 receptor agonists and resmetirom, confer parallel benefits on peripheral nerve structure and function as they improve adverse liver histological features.

## 5. Conclusions

This exploratory study suggests that adults with MASLD (even in the absence of T2D) may exhibit small nerve fibre abnormalities, with hepatic steatosis and metabolic dysfunction associated with measures of small nerve fibre morphology. Dietary weight loss was accompanied by reductions in liver fat, improvements in neuropathic symptoms and increased CNFD, although CNBD and CNFL did not change significantly. The association between changes in liver fat and CNFL provides preliminary evidence of a potential relationship linking hepatic and metabolic health to neural health. Larger, adequately powered prospective studies are required to confirm these findings and determine whether improvements in hepatic health directly influence peripheral nerve structure and function.

## Figures and Tables

**Table 1:** Baseline comparisons and delta change with dietary intervention in measured variables at 3 months.

Covariate	Baseline MASLD Median (IQR)	Baseline Controls Median (IQR)	*p* Value	3 Months Median (IQR)	Delta Change (IQR)	*p* Value
Demographics						
Age, years	48 (40, 53)	40 (33, 46)	0.10			
Sex, n	Male (5), Female (7)	Male (11) Female (5)				
Anthropometry						
Weight, kg	112.7 (87.4, 118.2)	71.0 (56.5, 79.8)	<0.01	100.9 (75.5, 105.7)	−12.5 (−13.6, −8.4)	<0.01
Body mass index kg/m^2^	35.7 (35.3, 37.8)	25.3 (22.9, 28.9)	<0.01	32.0 (30.3, 33.7)	−4.0 (−5.4, −3.0)	<0.01
Waist circumference, (cm)	114.3 (105.6, 120.5)	79.5 (72.5, 86.3)	<0.01	99.5 (93.1, 113.6)	−12.0 (−17.9, −5.6)	0.01
Hip circumference, (cm)	117.0 (115.3, 126.3)	84.5 (80.0, 90.5)	<0.01	109.0 (106.3, 118.4)	−8.8 (−9.9, −4.9)	0.02
Blood pressure, mmHg						
Systolic	130 (123, 148)	130 (120, 142)	0.91	120 (123, 148)	−5 (−19, 0)	0.03
Diastolic	81 (72, 89)	79 (72, 86)	0.45	78 (64, 88)	−1 (−8, 5.5)	0.26
Biochemistry						
Insulin resistance						
Fasting glucose, mmol/L	5.1 (4.5, 5.5)	4.2 (4.0, 4.4)	<0.01	4.75 (4.53, 5.05)	−0.20 (−0.60, 0.05)	0.05
HbA1c, mmol/mol	37 (35, 39)	34 (32, 37)	0.02	37 (36, 39)	0 (−1, 0)	0.47
Lipid profile, mmol/L						
Triglycerides	1.6 (1.2, 2.0)	1.8 (1.4, 2.5)	0.07	1.2 (1.1, 1.4)	−0.3 (−0.7, 0.0)	0.31
HDL cholesterol	1.1 (1.0, 1.5)	1.4 (1.0, 1.7)	0.35	1.1 (1.0, 1.5)	0.1 (−0.2, 0.2)	0.67
LDL cholesterol	3.8 (3.3, 4.2)	2.5 (2.1, 2.8)	<0.01	3.9 (2.9, 4.3)	−0.1 (−0.6, 0.4)	0.92
Total cholesterol	5.7 (5.5, 6.3)	4.4 (3.9, 4.8)	<0.01	5.7 (5.1, 5.8)	0.1 (−0.9, 0.2)	0.42
Liver function tests						
AST, u/L	19 (16, 30)	21 (19, 23)	0.31	18 (17, 25)	−4 (−11, 2)	0.23
ALT, u/L	23 (17, 39)	19 (13, 25)	0.09	22 (16, 28)	−2 (−23, 2)	0.18
Platelets, ×10^9^/L	270 (236, 312)	278 (218, 340)	0.87	265 (232, 311)	3 (−12, 6)	0.89
Albumin, g/L	45 (43, 47)	40 (38, 41)	<0.01	46 (44, 48)	1 (0, 3)	0.01
Risk stratification tools						
Cardiovascular						
QRISK3	2.25 (1.00, 4.95)					
MetS traits	3 (2, 4)	2 (1, 3)	<0.01	2 (1, 3)	−1 (−0, −2)	0.04
Liver						
FIB-4	0.75 (0.62, 0.86)	0.70 (0.59, 0.85)	0.96	0.74 (0.56, 0.91)	−0.05 (−0.15, 0.05)	0.33
FLI	88.5 (78.3, 96.8)	18.0 (6.7, 34.7)	<0.01	66.4 (38.8, 86.5)	−12.6 (−41.8, −6.8)	0.03
NFS	−2.00 (−2.56, −1.34)	−2.80 (−3.56, −1.89)	0.03	−2.08 (−2.82, −0.88)	−0.26 (−0.57, 0.07)	0.96
Imaging						
Transient elastography						
CAP (dB/m)	316 (262, 343)	209 (197, 236)	<0.01	255 (223, 283)	−8 (−106, 19)	0.16
Stiffness (kPa)	4.2 (3.5, 5.9)			5.1 (3.5, 5.6)	0.0 (−0.5, 0.6)	0.61
MRI liver						
PDFF (normal < 4.8%)	9.4 (3.8, 14.2)			4.4 (2.5, 6.1)	−5.0 (−8.2, −0.6)	0.03
cT1 (normal < 784 ms)	805 (736, 857)			766 (715, 820)	−30 (−76, 41)	0.29
MRI kidney						
Left	1356 (1263, 1386)			1341 (1323, 1357)	−27 (−39, 70)	0.28
Right	1301 (1267, 1364)			1339 (1319, 1362)	26 (15, 65)	0.09
MRI pancreas						
PDFF, % (normal < 4.6%)	3.5 (1.9, 6.8)			3.1 (2.2, 5.7)	−0.3 (−1.6, 0.9)	0.30
srT1 ROI, ms (normal < 803 ms)	755 (699, 801)			749 (727, 836)	6.5 (−23.8, 64.8)	0.31
Corneal confocal microscopy						
CNFD, fibres/mm^2^	25.70 (23.08, 28.90)	28.42 (26.56, 30.73)	0.03	28.61 (23.29, 32.45)	1.87 (−0.74, 6.25)	0.05
CNBD, branches/mm^2^	34.06 (30.47, 47.15)	37.94 (31.25, 45.83)	0.41	37.45 (25.68, 52.57)	0.16 (−5.14, 10.49)	0.42
CNFL, mm/mm^2^	14.09 (12.93, 15.63)	17.11 (15.89, 18.40)	0.02	15.44 (12.98, 16.90)	0.18 (−1.07, 2.47)	0.34
Neuropathy symptom questionnaires						
McGill Pain Questionnaire	1.50 (0.00, 3.25)			0.00 (0.00, 2.00)	−0.25 (−1.50, 0.00)	0.06
Neuropathy Disability Score	0.00 (0.00, 0.00)			0.00 (0.00, 0.00)	0.00 (0.00, 0.00)	0.62
Neuropathy Symptom Profile	2.00 (0.00, 6.75)			0.50 (0.00, 1.25)	−1.50 (−5.25, 0.00)	0.02
Pain Detect Score	6.00 (1.00, 8.00)			0.00 (0.00, 3.00)	−5.00 (−8.00, 0.00)	0.02
Small Fiber Neuropathy Screening List	12.00 (0.00, 32.50)			1.00 (0.00, 16.00)	−11.00 (−14.50, 0.00)	0.03

**Table 2:** Multivariable linear regression analyses, including age, body mass index (BMI), total cholesterol and transient elastography-derived controlled attenuation parameter (CAP), evaluating independent associations between strongly correlated variables during univariate analysis with corneal confocal microscopy metrics (corneal nerve fibre density, CNFD; corneal nerve branch density, CNBD; and corneal nerve fibre length, CNFL).

	CNFD				CNBD				CNFL			
	uβ	95% CI	sβ	*p*	uβ	95% CI	sβ	*p*	uβ	95% CI	sβ	*p*
Age, years	0.27	0.08, 0.46	0.57	0.01	0.54	−0.30, 1.37	0.35	0.19	0.08	−0.06, 0.22	0.26	0.24
BMI, kg/m^2^	−0.15	−0.43, 0.14	−0.24	0.29	−0.02	−1.24, 1.21	−0.01	0.98	−0.06	−0.26, 0.16	−0.13	0.59
Total cholesterol	−1.60	−3.33, 0.13	−0.37	0.07	−3.18	−10.60, 4.25	−0.23	0.38	−1.54	−2.81, −0.27	−0.53	0.02
CAP	−0.03	−0.06, −0.01	−0.45	<0.05	−0.03	−0.15, 0.09	−0.17	0.57	−0.01	−0.03, 0.01	−0.20	0.41

## Data Availability

The data presented in this study are available on request from the corresponding author due to ethical and participant confidentiality considerations.

## Supplementary Materials

The following supporting information can be downloaded at: https://www.mdpi.com/article/10.3390/nu18193242/s1, Figure S1: Boxplots demonstrating change in non-invasive tests of steatosis and fibrosis from baseline to 3 months; Figure S2: Boxplots demonstrating change in neuropathy symptom questionnaires from baseline to 3 months; Table S1: Univariate correlation analyses assessing the association between anthropometric, biochemical and non-invasive liver markers with corneal confocal microscopy metrics (corneal nerve fibre density, CNFD; corneal nerve branch density, CNBD; and corneal nerve fibre length, CNFL) in all participants (those with metabolic dysfunction-associated steatotic liver disease, and controls); Table S2: Spearman Rho baseline correlation analyses assessing the correlative associations between liver imaging and covariates; Table S3: Spearman Rho correlation analyses assessing the correlative associations between corneal confocal microscopy and covariates at baseline; Table S4: Spearman Rho correlation analyses assessing the correlative associations between delta change in liver imaging parameters (proton density fat fraction, liver-iron corrected T1) and covariates at 3 months; Table S5: Spearman Rho correlation analyses assessing the correlative associations between delta change in corneal confocal microscopy parameters and covariates at 3 months; Figure S3: Scatter plots with trend lines demonstrating the significant results of Spearman Rho correlation analyses assessing the correlative associations between corneal confocal microscopy and covariates at baseline; Figure S4: Scatter plots with trend lines demonstrating the significant results of Spearman Rho correlation analyses assessing the correlative associations between delta change in neuropathy measures, including corneal confocal microscopy and questionnaires, and delta changes liver health (assessed via MRI), at 3 months.

## References

[1] Younossi Z.M., Kalligeros M., Henry L. (2025). Epidemiology of metabolic dysfunction-associated steatotic liver disease. Clin. Mol. Hepatol..

[2] Cusi K., Abdelmalek M.F., Apovian C.M., Balapattabi K., Bannuru R.R., Barb D., Bardsley J.K., Beverly E.A., Corbin K.D., ElSayed N.A. (2025). Metabolic Dysfunction-Associated Steatotic Liver Disease (MASLD) in People With Diabetes: The Need for Screening and Early Intervention. A Consensus Report of the American Diabetes Association. Diabetes Care.

[3] Henney A.E., Riley D.R., Hydes T.J., Anson M., Ibarburu G.H., Zhao S.S., Cuthbertson D.J., Alam U. (2024). Metabolic syndrome traits differentially and cumulatively influence micro- and macrovascular disease risk in patients with MASLD. Liver Int..

[4] Cuthbertson D.J., Kennedy O.J., Bilson J., Hydes T.J., Targher G., Glyn-Owen K., Buchanan R., Roderick P., Byrne C.D. (2025). Impact of metabolic dysfunction severity in steatotic liver disease and its interaction with liver fibrosis on all-cause mortality and multiple hepatic and extra-hepatic outcomes. Metabolism.

[5] Deravi N., Firouzabadi F.D., Moosaie F., Asadigandomani H., Bafrani M.A., Yoosefi N., Poopak A., Firouzabadi M.D., Poudineh M., Rabizadeh S. (2023). Non-alcoholic fatty liver disease and incidence of microvascular complications of diabetes in patients with type 2 diabetes: A prospective cohort study. Front. Endocrinol..

[6] Xie J., Zhu Q., Liu X., Qu Y., Jiang Y., Wei J., Xu Y., Liu K. (2026). Association of metabolic dysfunction-associated fatty liver disease with microvascular complications in adults with prediabetes. Nutr. Metab. Cardiovasc. Dis..

[7] Trivedi H.D., Tran Q., Fricker Z., Curry M.P., Li J.X., Lai M. (2023). Type 2 diabetes complications are associated with liver fibrosis independent of hemoglobin A1c. Ann. Hepatol..

[8] Gu X., Tang D., Xuan Y., Shen Y., Lu L.Q. (2023). Association between nonalcoholic fatty liver disease and peripheral neuropathy in US population, a cross-sectional study. Sci. Rep..

[9] Huang J., Li R., Liu N., Yi N., Zheng H., Zhang Q., Zhou L., Zhou L., Hu R., Lu B. (2021). Liver fibrosis is independently associated with diabetic peripheral neuropathy in type 2 diabetes mellitus. J. Diabetes Investig..

[10] Kim K., Oh T.J., Cho H.C., Lee Y.K., Ahn C.H., Koo B.K., Moon J.H., Choi S.H., Jang H.C. (2021). Liver fibrosis indices are related to diabetic peripheral neuropathy in individuals with type 2 diabetes. Sci. Rep..

[11] Hurr C., Simonyan H., Morgan D.A., Rahmouni K., Young C.N. (2019). Liver sympathetic denervation reverses obesity-induced hepatic steatosis. J. Physiol..

[12] FDA (2024). FDA Approves First Treatment for Patients with Liver Scarring Due to Fatty Liver Disease.

[13] Huh Y.J. (2025). Cost-Effectiveness of Obesity Treatments: Glucagon-Like Peptide-1 Receptor Agonists, Endoscopic Sleeve Gastroplasty, and Metabolic/Bariatric Surgery. J. Metab. Bariatr. Surg..

[14] European Association for the Study of the Liver (EASL), European Association for the Study of Diabetes (EASD), European Association for the Study of Obesity (EASO) (2024). EASL-EASD-EASO Clinical Practice Guidelines on the management of metabolic dysfunction-associated steatotic liver disease (MASLD). J. Hepatol..

[15] Vilar-Gomez E., Martinez-Perez Y., Calzadilla-Bertot L., Torres-Gonzalez A., Gra-Oramas B., Gonzalez-Fabian L., Friedman S.L., Diago M., Romero-Gomez M. (2015). Weight Loss Through Lifestyle Modification Significantly Reduces Features of Nonalcoholic Steatohepatitis. Gastroenterology.

[16] Dobbie L.J., Burgess J., Hamid A., Nevitt S.J., Hydes T.J., Alam U., Cuthbertson D.J. (2024). Effect of a Low-Calorie Dietary Intervention on Liver Health and Body Weight in Adults with Metabolic-Dysfunction Associated Steatotic Liver Disease (MASLD) and Overweight/Obesity: A Systematic Review and Meta-Analysis. Nutrients.

[17] Taylor R., Al-Mrabeh A., Sattar N. (2019). Understanding the mechanisms of reversal of type 2 diabetes. Lancet Diabetes Endocrinol..

[18] Adam S., Azmi S., Ho J.H., Liu Y., Ferdousi M., Siahmansur T., Kalteniece A., Marshall A., Dhage S.S., Iqbal Z. (2021). Improvements in Diabetic Neuropathy and Nephropathy After Bariatric Surgery: A Prospective Cohort Study. Obes. Surg..

[19] Azmi S., Ferdousi M., Liu Y., Adam S., Iqbal Z., Dhage S., Ponirakis G., Siahmansur T., Marshall A., Petropoulos I. (2021). Bariatric surgery leads to an improvement in small nerve fibre damage in subjects with obesity. Int. J. Obes..

[20] Lazarus J.V., Newsome P.N., Francque S.M., Kanwal F., Terrault N.A., Rinella M.E. (2023). A multisociety Delphi consensus statement on new fatty liver disease nomenclature. J. Hepatol..

[21] Diamond C., Pansini M., Hamid A., Eichert N., Pandya P., Ali S.N., Kemp G.J., Thanabalasingham G., Brears H.T., Cuthbertson D.J. (2024). Quantitative Imaging Reveals Steatosis and Fibroinflammation in Multiple Organs in People With Type 2 Diabetes: A Real-World Study. Diabetes.

[22] Tavakoli M., Ferdousi M., Petropoulos I.N., Morris J., Pritchard N., Zhivov A., Ziegler D., Pacaud D., Romanchuk K., Perkins B.A. (2015). Normative values for corneal nerve morphology assessed using corneal confocal microscopy: A multinational normative data set. Diabetes Care.

[23] Lee J., Vali Y., Boursier J., Spijker R., Anstee Q.M., Bossuyt P.M., Zafarmand M.H. (2021). Prognostic accuracy of FIB-4, NAFLD fibrosis score and APRI for NAFLD-related events: A systematic review. Liver Int..

[24] Iqbal Z., Kalteniece A., Ferdousi M., Adam S., D’ONofrio L., Ho J.H., Rao A.P., Dhage S., Azmi S., Liu Y. (2021). Corneal Keratocyte Density and Corneal Nerves Are Reduced in Patients With Severe Obesity and Improve After Bariatric Surgery. Investig. Ophthalmol. Vis. Sci..

[25] Callaghan B.C., Reynolds E.L., Banerjee M., Akinci G., Chant E., Villegas-Umana E., Rothberg A.E., Burant C.F., Feldman E.L. (2021). Dietary weight loss in people with severe obesity stabilizes neuropathy and improves symptomatology. Obesity.

[26] Azmi S., Jeziorska M., Ferdousi M., Petropoulos I.N., Ponirakis G., Marshall A., Alam U., Asghar O., Atkinson A., Jones W. (2019). Early nerve fibre regeneration in individuals with type 1 diabetes after simultaneous pancreas and kidney transplantation. Diabetologia.

[27] Cuthbertson D.J., Keating S.E., Pugh C.J.A., Owen P.J., Kemp G.J., Umpleby M., Geyer N.G., Chinchilli V.M., Stine J.G. (2024). Exercise improves surrogate measures of liver histological response in metabolic dysfunction-associated steatotic liver disease. Liver Int..

[28] Ponirakis G., Abdul-Ghani M.A., Jayyousi A., Almuhannadi H., Petropoulos I.N., Khan A., Gad H., Migahid O., Megahed A., DeFronzo R. (2020). Effect of treatment with exenatide and pioglitazone or basal-bolus insulin on diabetic neuropathy: A substudy of the Qatar Study. BMJ Open Diabetes Res. Care.

[29] Adhikari U., Gad H., Chatterjee D., Malhotra C., Bhadada S.K., Malik R.A., Rastogi A. (2025). Dapagliflozin for Small Nerve Fibre Regeneration in Diabetic Peripheral Neuropathy: A Randomised Controlled Study (DINE). J. Peripher. Nerv. Syst..

[30] Adam S., Ho J.H., Liu Y., Siahmansur T., Siddals K., Iqbal Z., Azmi S., Senapati S., New J., Jeziorska M. (2022). Bariatric Surgery-induced High-density Lipoprotein Functionality Enhancement Is Associated With Reduced Inflammation. J. Clin. Endocrinol. Metab..

[31] Azmi S., Ferdousi M., Liu Y., Adam S., Siahmansur T., Ponirakis G., Marshall A., Petropoulos I.N., Ho J.H., Syed A.A. (2021). The role of abnormalities of lipoproteins and HDL functionality in small fibre dysfunction in people with severe obesity. Sci. Rep..

[32] Marra F., Svegliati-Baroni G. (2018). Lipotoxicity and the gut-liver axis in NASH pathogenesis. J. Hepatol..

[33] Neuschwander-Tetri B.A. (2010). Hepatic lipotoxicity and the pathogenesis of nonalcoholic steatohepatitis: The central role of nontriglyceride fatty acid metabolites. Hepatology.

[34] Coppey L., Davidson E., Shevalye H., Obrosov A., Torres M., Yorek M.A. (2020). Progressive Loss of Corneal Nerve Fibers and Sensitivity in Rats Modeling Obesity and Type 2 Diabetes Is Reversible with Omega-3 Fatty Acid Intervention: Supporting Cornea Analyses as a Marker for Peripheral Neuropathy and Treatment. Diabetes Metab. Syndr. Obes..

[35] Yagihashi S., Mizukami H., Sugimoto K. (2011). Mechanism of diabetic neuropathy: Where are we now. and where to go?. J. Diabetes Investig..

[36] Burgess J., Frank B., Marshall A., Khalil R.S., Ponirakis G., Petropoulos I.N., Cuthbertson D.J., Malik R.A., Alam U. (2021). Early Detection of Diabetic Peripheral Neuropathy: A Focus on Small Nerve Fibres. Diagnostics.

[37] Smith A.G., Singleton J.R. (2013). Obesity and hyperlipidemia are risk factors for early diabetic neuropathy. J. Diabetes Complicat..

[38] Bashir B., Pasha R., Kamath A., Malik R.A., Ferdousi M., Soran H. (2025). Small nerve fibre damage and cardiac autonomic dysfunction in patients with hypertriglyceridaemia. Atherosclerosis.

[39] Bashir B., Pasha R., Kamath A., Wang J., Malik R.A., Hegele R.A., Ferdousi M., Soran H. (2025). Neurodegeneration in familial chylomicronemia syndrome. J. Clin. Lipidol..

[40] D’ONofrio L., Ferdousi M., Kalteniece A., Iqbal Z., Petropoulos I.N., Ponirakis G., Buzzetti R., Malik R.A., Soran H. (2022). Corneal confocal microscopy identifies small nerve fibre damage in patients with hypertriglyceridemia. J. Clin. Lipidol..

[41] Ponirakis G., Peerzada L., Petropoulos I.N., Gad H., Abdulshakoor S., Concepcion J.M., Khalfalla S.H., Elamin I.S.A., AlZawqari A.T.H., Elgassim E. (2025). Differential Effects of Visceral and Subcutaneous Adiposity on Peripheral Neuropathy. J. Peripher. Nerv. Syst..

[42] Kalteniece A., Ferdousi M., Azmi S., Mubita W.M., Marshall A., Lauria G., Faber C.G., Soran H., Malik R.A. (2020). Corneal confocal microscopy detects small nerve fibre damage in patients with painful diabetic neuropathy. Sci. Rep..

[43] Angarita-Rodríguez A., Matiz-González J.M., Pinzón A., Aristizabal A.F., Ramírez D., Barreto G.E., González J. (2024). Enzymatic Metabolic Switches of Astrocyte Response to Lipotoxicity as Potential Therapeutic Targets for Nervous System Diseases. Pharmaceuticals.

[44] Kiuchi M.G., Carnagarin R., Matthews V.B., Schlaich M.P. (2023). Multi-organ denervation: A novel approach to combat cardiometabolic disease. Hypertens. Res..

[45] Hwang J., Okada J., Liu L., Pessin J.E., Schwartz G.J., Jo Y.-H. (2024). The development of hepatic steatosis depends on the presence of liver-innervating parasympathetic cholinergic neurons in mice fed a high-fat diet. PLoS Biol..

