# Supplementary material for: Small-Nerve Fibre Pathology in Metabolic Dysfunction-Associated Steatotic Liver Disease Is Associated with Liver Fat/Inflammation and Improves with Weight Loss
Source: Nutrients. 2026 Oct 1;18(19):3242. doi: 10.3390/nu18193242 (PMC13666698; doi:10.3390/nu18193242)
Supplement: Supplementary file 1 [file nutrients-18-03242-s001.zip › nutrients-4525006-supplementary.pdf]

Supplementary Material Improvements in small-nerve fibre pathology parallel reductions in liver steatosis and fibro inflammation in adults with metabolic dysfunction-associated steatotic liver disease with diet-induced weight loss.

Contents

- ***Page 2:*** **Supplementary Material Figure S1** Boxplots demonstrating change in non-invasive markers of steatosis and fibrosis from baseline to 3 months.
- ***Page 3:*** **Supplementary Material Figure S2** Boxplots demonstrating change in neuropathy symptom questionnaires from baseline to 3 months.
- ***Pages 4-5:*** **Supplementary Material Table S1** Univariate correlation analyses assessing the association between anthropometric, biochemical and non-invasive liver markers with corneal confocal microscopy in all participants.
- ***Pages 6-7:*** **Supplementary Material Table S2** Univariate correlation analyses assessing the correlative associations between liver imaging and covariates.
- ***Page 8:*** **Supplementary Material Table S3** Univariate correlation analyses assessing the correlative associations between corneal confocal microscopy and covariates at baseline.
- ***Pages 9-10:*** **Supplementary Material Table S3** Univariate correlation analyses assessing the correlative associations between delta change in liver imaging parameters (proton density fat fraction, liver-iron corrected T1) and covariates at 3 months.
- ***Page 11:*** **Supplementary Material Table S4** Univariate correlation analyses assessing the correlative associations between delta change in corneal confocal microscopy parameters and covariates at 3 months.

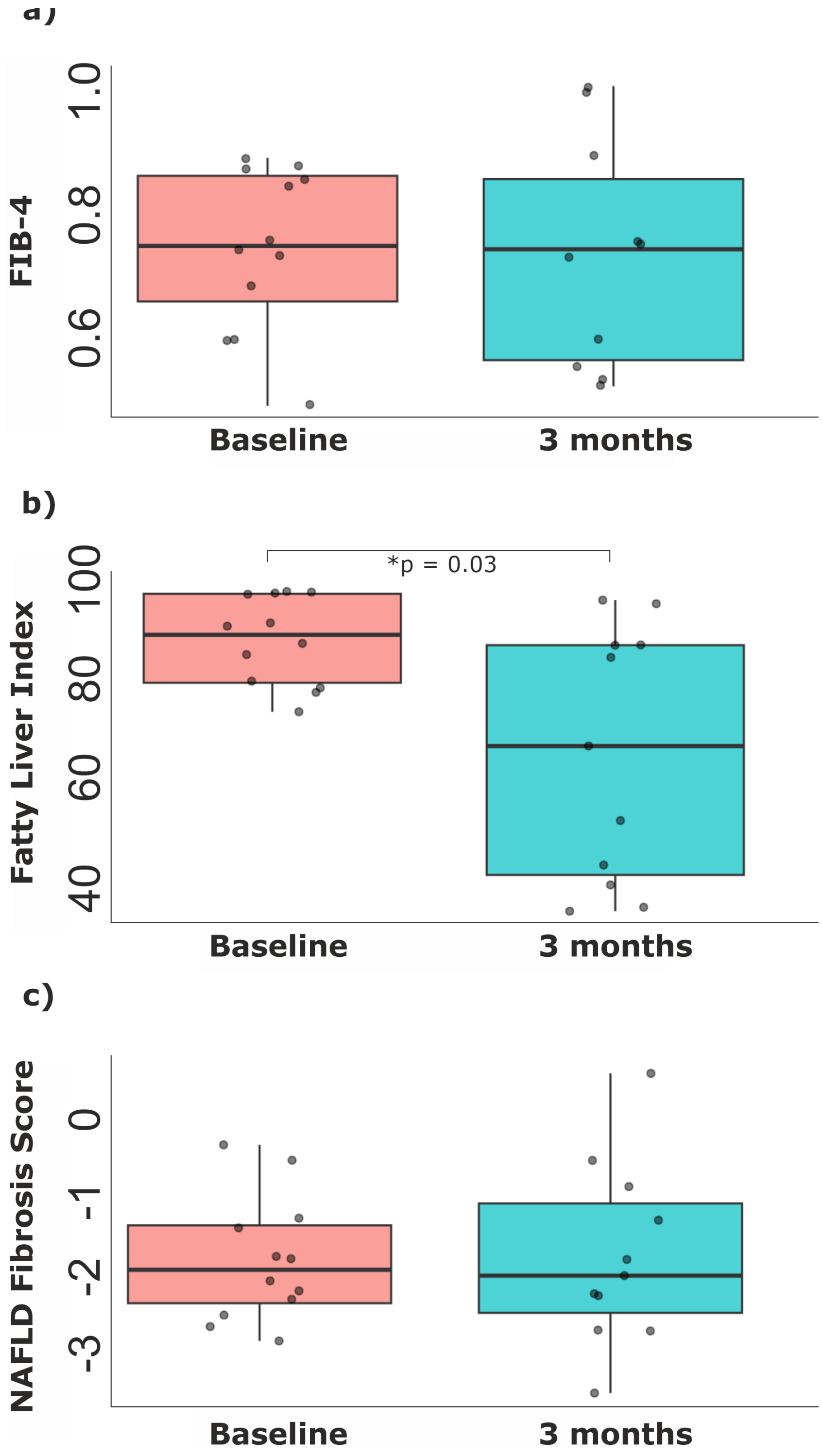

Supplementary Material Figure S1. Boxplots demonstrating change in non-invasive tests of steatosis and fibrosis from baseline to 3 months.

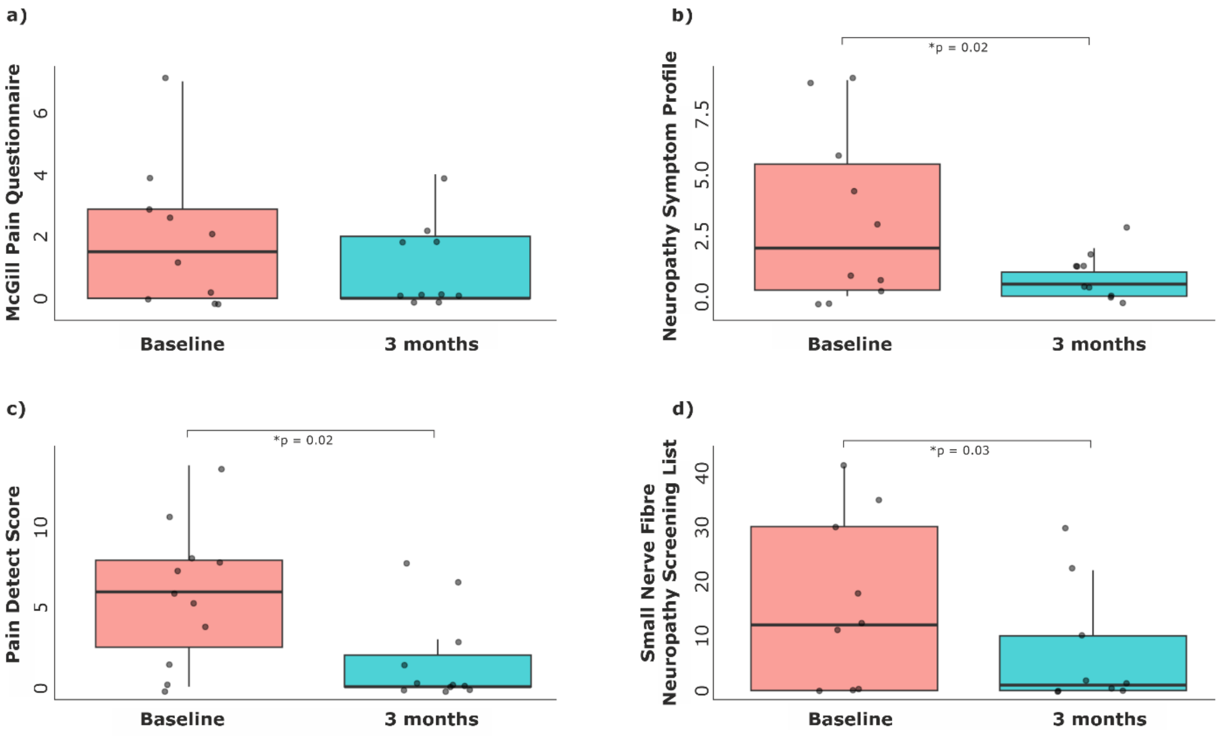

Supplementary Material Figure S2. Boxplots demonstrating change in neuropathy symptom questionnaires from baseline to 3 months.

Covariates	CNFD		CNBD		CNFL	
	Correlation coefficient	P value	Correlation coefficient	P value	Correlation coefficient	P value
**Anthropometrics**						
BMI, kg/m^2	**-0.42**	**0.03***	-0.07	0.72	**-0.40**	**0.03***
Weight, kg	**-0.53**	**<0.01***	-0.09	0.65	**-0.44**	**0.02***
Waist circumference, cm	**-0.45**	**0.02***	-0.01	0.98	-0.36	0.06
Hip circumference, cm	**-0.48**	**0.01***	-0.15	0.43	**-0.44**	**0.02***
Systolic blood pressure, mmHg	0.03	0.88	-0.15	0.45	-0.09	0.65
Diastolic blood pressure, mmHg	-0.13	0.50	-0.20	0.30	-0.15	0.46
**Biochemistry**						
Insulin resistance						
Fasting glucose	-0.24	0.22	-0.08	0.69	-0.30	0.13
HbA1c	-0.31	0.11	-0.34	0.08	-0.34	0.08
Lipid profile						
Triglycerides	0.20	0.31	-0.13	0.52	0.16	0.42
HDL cholesterol	-0.15	0.44	-0.24	0.22	-0.14	0.49
LDL cholesterol	-0.33	0.09	-0.08	0.68	**-0.52**	**0.01***
Total cholesterol	**-0.40**	**0.04***	-0.13	0.52	**-0.54**	**<0.01***
Liver function						
ALT	-0.23	0.23	-0.04	0.83	-0.12	0.55
AST	-0.12	0.54	0.02	0.93	-0.02	0.93
Platelets	0.12	0.55	0.35	0.07	0.29	0.13
Albumin	-0.34	0.07	-0.12	0.54	-0.25	0.21
**Risk stratification scores**						
MetS components	-0.20	0.31	-0.20	0.32	-0.28	0.16
Liver steatosis and fibrosis scores						
FIB-4	0.07	0.74	-0.07	0.72	-0.13	0.50
Fatty Liver Index	-0.42	0.03	-0.10	0.62	**-0.41**	**0.04***

NAFLD Fibrosis Score	-0.15	0.44	-0.12	0.55	-0.30	0.12
**Fibroscan**						
CAP	**-0.45**	**0.02***	-0.11	0.58	**-0.40**	**0.04***

Supplementary Material Table S1. Univariate correlation analyses assessing the association between anthropometric, biochemical and non-invasive liver markers with corneal confocal microscopy metrics (corneal nerve fibre density, CNFD; corneal nerve branch density, CNBD; and corneal nerve fibre length, CNFL) in all participants (those with metabolic dysfunction-associated steatotic liver disease, and controls).

Covariates	**Liver PDFF**		**Liver-iron cT1**	
	Correlation coefficient	P value	Correlation coefficient	P value
**Anthropometrics**				
BMI, kg/m^2	0.24	0.45	0.51	0.09
Weight, kg	0.33	0.29	0.11	0.74
Waist circumference, cm	0.22	0.50	0.31	0.33
Hip circumference, cm	-0.24	0.46	0.07	0.84
Systolic blood pressure, mmHg	0.31	0.33	0.25	0.44
Diastolic blood pressure, mmHg	-0.08	0.81	0.11	0.75
**Biochemistry**				
Insulin resistance				
Fasting glucose	-0.18	0.59	-0.21	0.51
HbA1c	0.53	0.10	0.13	0.71
Lipid profile				
Triglycerides	0.46	0.14	0.35	0.27
HDL cholesterol	-0.43	0.16	-0.37	0.23
**Risk stratification scores**				
QRISK3	0.36	0.25	0.40	0.19
MetS components	0.24	0.45	0.15	0.65
Liver steatosis and fibrosis scores				
FIB-4	0.28	0.38	-0.27	0.40
Fatty Liver Index	0.44	0.15	**0.58**	**0.04***
NAFLD Fibrosis Score	0.34	0.29	0.04	0.90
**Fibroscan**				
CAP	0.24	0.48	-0.11	0.74
Stiffness	0.51	0.11	0.35	0.29
**Corneal confocal microscopy**				
CNFD	-0.21	0.51	-0.17	0.59
CNBD	-0.36	0.25	0.19	0.56
CNFL	-0.18	0.57	0.15	0.63
**Neuropathy questionnaires**				

McGill Pain Questionnaire	0.09	0.81	0.29	0.41
Neuropathy Disability Score	-0.28	0.40	0.39	0.23
Neuropathy Symptom Profile	0.04	0.92	0.30	0.40
Pain Detect Score	0.11	0.74	0.34	0.31
Small Fiber Neuropathy Screening List	0.22	0.57	0.42	0.26

Supplementary Material Table S2. Spearman Rho baseline correlation analyses assessing the correlative associations between liver imaging and covariates.

Covariates	CNFD		CNBD		CNFL	
	Correlation coefficient	P value	Correlation coefficient	P value	Correlation coefficient	P value
**Anthropometrics**						
BMI, kg/m^2	**-0.64**	**0.02***	-0.22	0.49	-0.45	0.15
Weight, kg	**-0.61**	**0.04***	-0.25	0.44	-0.48	0.11
Waist circumference, cm	**-0.59**	**0.04***	0.08	0.81	-0.18	0.59
Hip circumference, cm	**-0.57**	**0.05***	-0.26	0.41	-0.51	0.09
Systolic blood pressure, mmHg	-0.38	0.23	-0.41	0.18	-0.48	0.12
Diastolic blood pressure, mmHg	**-0.66**	**0.02***	-0.45	0.15	**-0.62**	**0.03***
**Biochemistry**						
Insulin resistance						
Fasting glucose	0.27	0.39	-0.21	0.52	0.20	0.54
HbA1c	-0.33	0.32	-0.58	0.06	-0.39	0.24
Lipid profile						
Triglycerides	0.27	0.39	0.07	0.84	0.33	0.29
HDL cholesterol	-0.30	0.35	-0.22	0.50	-0.44	0.15
**Risk stratification scores**						
QRISK3	0.03	0.94	-0.21	0.51	-0.12	0.72
MetS components	0.02	0.95	-0.21	0.51	-0.01	0.98
Liver steatosis and fibrosis scores						
FIB-4	-0.08	0.82	**-0.61**	**0.04***	-0.48	0.12
Fatty Liver Index	-0.36	0.24	-0.18	0.57	-0.05	0.88
NAFLD Fibrosis Score	-0.01	0.97	**-0.58**	**0.05***	-0.34	0.28
**Fibroscan**						
CAP	-0.32	0.35	-0.35	0.30	-0.28	0.40
Stiffness	0.07	0.84	-0.14	0.69	0.18	0.60

Supplementary Material Table S3. Spearman Rho correlation analyses assessing the correlative associations between corneal confocal microscopy and covariates at baseline.

Covariates	**Liver PDFF**		**Liver-iron cT1**	
	Correlation coefficient	P value	Correlation coefficient	P value
**Anthropometrics**				
BMI, kg/m^2	0.48	0.12	0.48	0.11
Weight, kg	0.38	0.23	0.52	0.08
Waist circumference, cm	0.31	0.34	0.40	0.19
Hip circumference, cm	0.15	0.63	0.08	0.81
Systolic blood pressure, mmHg	0.14	0.67	0.38	0.22
Diastolic blood pressure, mmHg	0.30	0.35	**0.60**	**0.04***
**Biochemistry**				
Insulin resistance				
Fasting glucose	-0.41	0.19	-0.03	0.92
HbA1c	0.26	0.43	-0.01	0.98
Lipid profile				
Triglycerides	**0.73**	**0.01***	0.21	0.55
HDL cholesterol	-0.26	0.43	-0.43	0.19
**Risk stratification scores**				
Liver steatosis and fibrosis scores				
FIB-4	0.12	0.75	0.06	0.87
Fatty Liver Index	0.46	0.16	0.26	0.45
NAFLD Fibrosis Score	0.45	0.17	0.31	0.36
**Fibroscan**				
CAP	0.01	0.97	0.03	0.93
Stiffness	0.42	0.20	0.49	0.13
**Corneal confocal microscopy**				
CNFD	-0.47	0.13	-0.44	0.16
CNBD	-0.41	0.18	-0.11	0.73

CNFL	**-0.63**	**0.03***	-0.14	0.67
**Neuropathy questionnaires**				
McGill Pain Questionnaire	0.26	0.47	0.19	0.60
Neuropathy Disability Score	0.10	0.76	**0.62**	**0.04***
Neuropathy Symptom Profile	-0.49	0.16	-0.36	0.31
Pain Detect Score	-0.40	0.23	-0.26	0.44
Small Fiber Neuropathy Screening List	0.30	0.44	**0.67**	**0.05***

Supplementary Material Table S4. Spearman Rho correlation analyses assessing the correlative associations between delta change in liver imaging parameters (proton density fat fraction, liver-iron corrected T1) and covariates at 3 months.

Covariates	**CNFD**		**CNBD**		**CNFL**	
	Correlation coefficient	P value	Correlation coefficient	P value	Correlation coefficient	P value

**Anthropometrics**						
BMI, kg/m^2	-0.35	0.27	-0.22	0.50	-0.91	0.78
Weight, kg	-0.33	0.30	0.26	0.41	0.14	0.66
Waist circumference, cm	-0.38	0.23	0.10	0.75	0.04	0.90
Hip circumference, cm	-0.33	0.30	0.32	0.31	0.16	0.62
Systolic blood pressure, mmHg	-0.38	0.23	0.41	0.19	0.01	0.98
Diastolic blood pressure, mmHg	**-0.72**	**0.01***	-0.03	0.94	-0.29	0.37
**Biochemistry**						
Insulin resistance						
Fasting glucose	-0.08	0.81	0.22	0.49	0.05	0.87
HbA1c	0.03	0.93	-0.46	0.16	-0.20	0.55
Lipid profile						
Triglycerides	-0.14	0.68	-0.40	0.23	-0.53	0.09
HDL cholesterol	0.12	0.73	-0.08	0.81	0.24	0.47
**Risk stratification scores**						
Liver steatosis and fibrosis scores						
FIB-4	-0.03	0.93	**-0.64**	**0.05***	-0.27	0.46
Fatty Liver Index	-0.42	0.20	-0.13	0.71	-0.23	0.50
NAFLD Fibrosis Score	-0.25	0.46	0.13	0.71	-0.23	0.50

Supplementary Material Table S5. Spearman Rho correlation analyses assessing the correlative associations between delta change in corneal confocal microscopy parameters and covariates at 3 months.

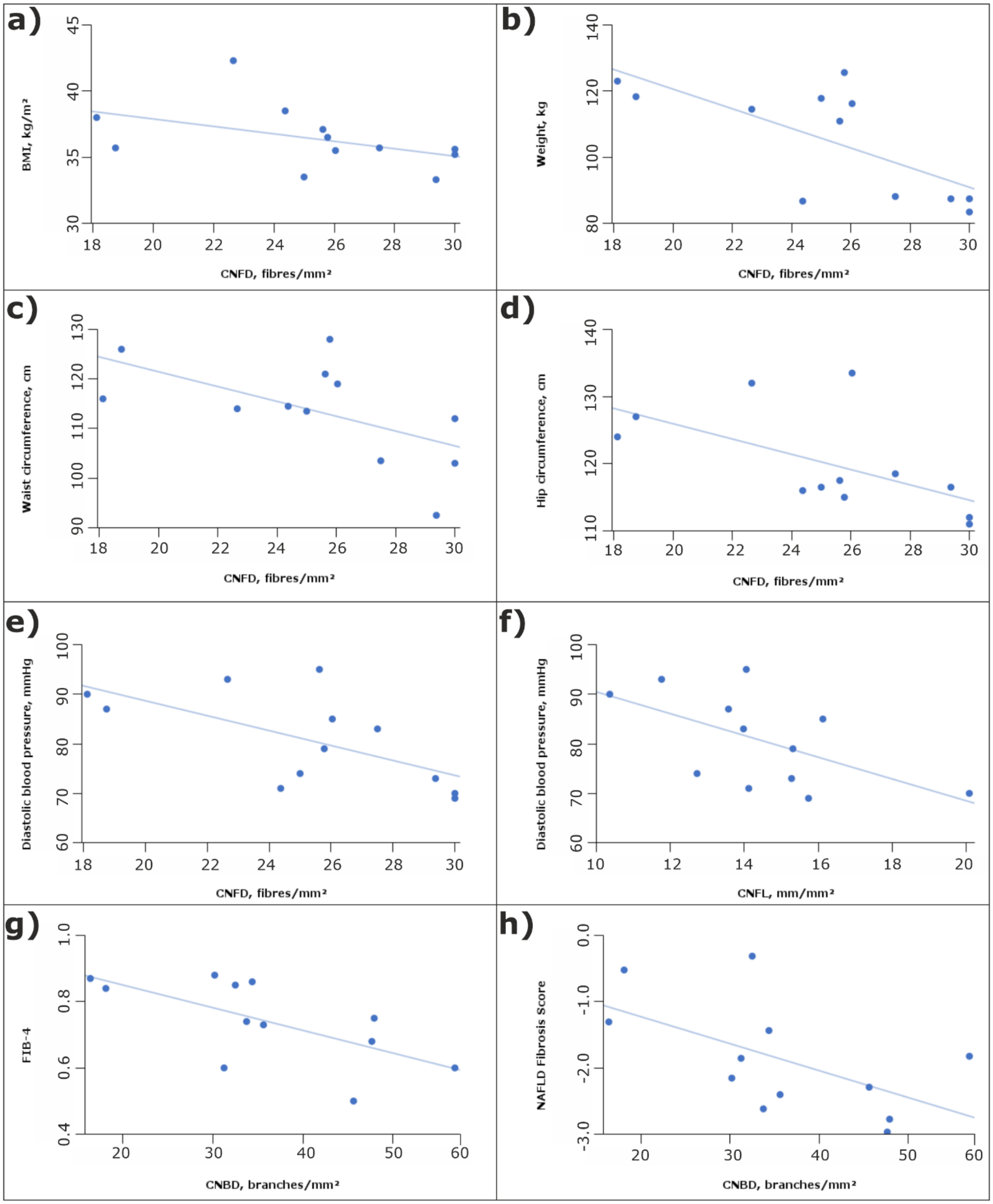

Supplementary Material Figure S3. Scatter plots with trend lines demonstrating the significant results of Spearman Rho correlation analyses assessing the correlative associations between corneal confocal microscopy and covariates at baseline.

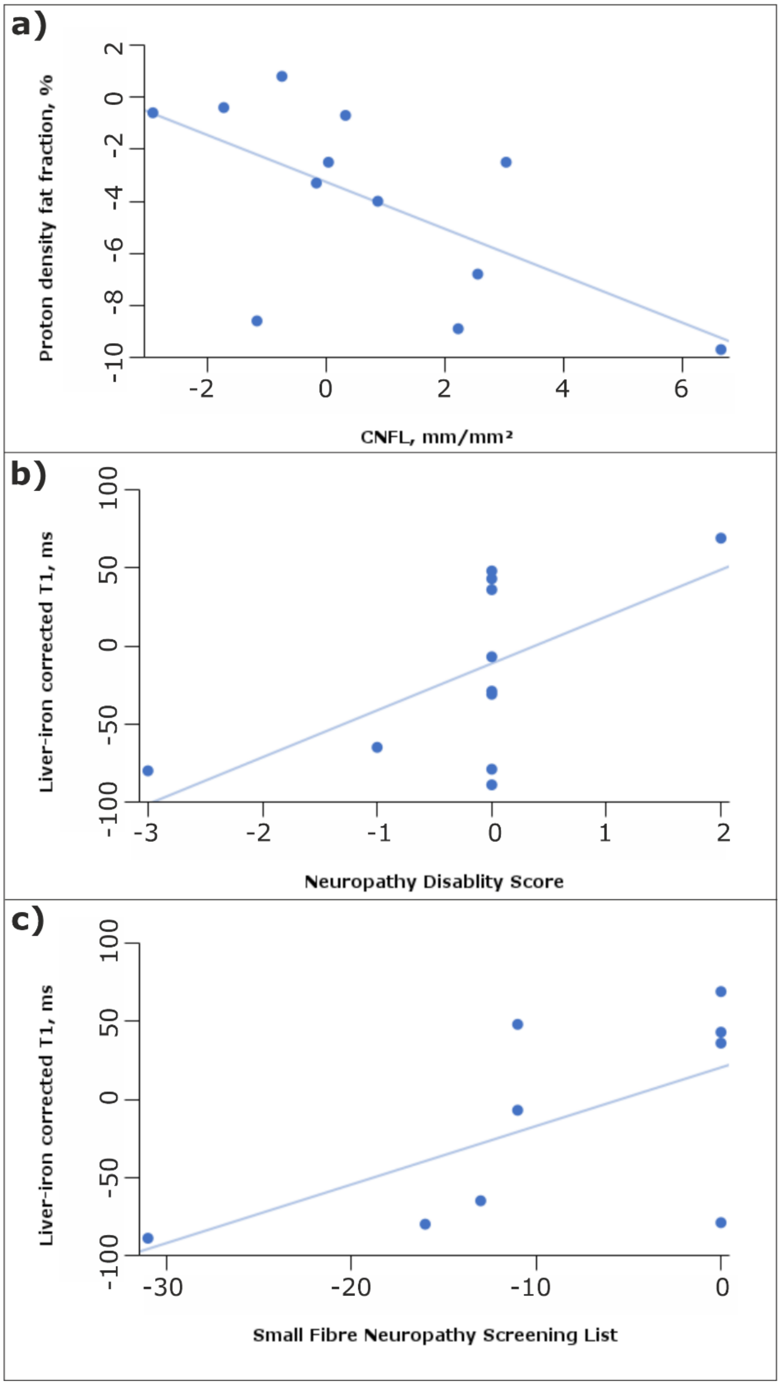

Supplementary Material Figure S4. Scatter plots with trend lines demonstrating the significant results of Spearman Rho correlation analyses assessing the correlative associations between delta change in neuropathy measures, including corneal confocal microscopy and questionnaires, and delta changes liver health (assessed via MRI), at 3 months.
